# Regulation of Heat Exchange across the Hornbill Beak: Functional Similarities with Toucans?

**DOI:** 10.1371/journal.pone.0154768

**Published:** 2016-05-18

**Authors:** T. M. F. N. van de Ven, R. O. Martin, T. J. F. Vink, A. E. McKechnie, S. J. Cunningham

**Affiliations:** 1 Percy FitzPatrick Institute of African Ornithology, DST-NRF Centre of Excellence, University of Cape Town, Cape Town, 7701, South Africa; 2 Institute for Coastal and Marine Research, Department of Botany, Nelson Mandela Metropolitan University, Port Elizabeth, 6031, South Africa; 3 DST-NRF Centre of Excellence at the Percy FitzPatrick Institute, Department of Zoology and Entomology, University of Pretoria, Pretoria, 0002, South Africa; St. Joseph's Hospital and Medical Center, UNITED STATES

## Abstract

Beaks are increasingly recognised as important contributors to avian thermoregulation. Several studies supporting Allen’s rule demonstrate how beak size is under strong selection related to latitude and/or air temperature (T_a_). Moreover, active regulation of heat transfer from the beak has recently been demonstrated in a toucan (*Ramphastos toco*, Ramphastidae), with the large beak acting as an important contributor to heat dissipation. We hypothesised that hornbills (Bucerotidae) likewise use their large beaks for non-evaporative heat dissipation, and used thermal imaging to quantify heat exchange over a range of air temperatures in eighteen desert-living Southern Yellow-billed Hornbills (*Tockus leucomelas*). We found that hornbills dissipate heat via the beak at air temperatures between 30.7°C and 41.4°C. The difference between beak surface and environmental temperatures abruptly increased when air temperature was within ~10°C below body temperature, indicating active regulation of heat loss. Maximum observed heat loss via the beak was 19.9% of total non-evaporative heat loss across the body surface. Heat loss per unit surface area via the beak more than doubled at T_a_ > 30.7°C compared to T_a_ < 30.7°C and at its peak dissipated 25.1 W m^-2^. Maximum heat flux rate across the beak of toucans under comparable convective conditions was calculated to be as high as 61.4 W m^-2^. The threshold air temperature at which toucans vasodilated their beak was lower than that of the hornbills, and thus had a larger potential for heat loss at lower air temperatures. Respiratory cooling (panting) thresholds were also lower in toucans compared to hornbills. Both beak vasodilation and panting threshold temperatures are potentially explained by differences in acclimation to environmental conditions and in the efficiency of evaporative cooling under differing environmental conditions. We speculate that non-evaporative heat dissipation may be a particularly important mechanism for animals inhabiting humid regions, such as toucans, and less critical for animals residing in more arid conditions, such as Southern Yellow-billed Hornbills. Alternatively, differences in beak morphology and hardness enforced by different diets may affect the capacity of birds to use the beak for non-evaporative heat loss.

## Introduction

There is increasing evidence for the importance of beaks in avian thermoregulation [1], with the beak identified as a significant avenue of non-evaporative heat dissipation in a number of species [2–5]. Variation in beak size among individuals has been shown to correspond with the thermal environment during development [6] and interspecific variation in beak size is related to environmental variables (maximum temperature, wind exposure, fresh water availability and thermal gradients) [2,7]. Adult Toco Toucans, *Ramphastos toco*, are able to vasodilate their extremely large beaks depending on thermal conditions, allowing heat to be dissipated from the beak. In this species, non-evaporative heat loss via the beak averages 60% of total non-evaporative heat loss at air temperatures (T_a_) above 28°C [4]. At air temperatures equivalent to 20–25°C below normothermic body temperature (T_b_), vasodilation of the networks below the rhamphotheca (the sheath of keratin that forms the outer surface of the beak), cause an increase in beak surface temperature [8]. Under these conditions, the beak acts as a heat radiator, reducing the need for evaporative heat dissipation. Toucans typically inhabit tropical forest environments [9], where ambient water vapour pressures reduce the potential for evaporative heat loss, likely promoting the relative contribution of non-evaporative heat dissipation to body temperature regulation. On the other hand, reduced reliance on evaporative heat dissipation probably also has an adaptive significance for water conservation in arid environments [2].

Hornbills (Bucerotiformes: Bucerotidae) are widespread in the Afrotropical and Indomalayan regions, with members of this taxon occupying habitats ranging from arid savannas to humid tropical forests [10]. Like toucans (Piciformes: Ramphastidae), hornbills have disproportionately large beaks and are a candidate for a similar capacity of heat exchange (however see Hughes [11]). Large beaks might be beneficial in various ways (dietary, thermoregulatory, sexual selection, etc.), however there must be a limit to the size of the beak due to the risk of heat uptake when T_a_ exceeds T_b_ [2]. Non-evaporative mechanisms of heat loss might be expected to be particularly important in environments where water is scarce and hence water-conservation critical. Southern Yellow-billed Hornbills (*Tockus leucomelas* Lichtenstein 1842) inhabiting the Kalahari Desert may be under strong selective pressure to conserve water, particularly during the summer breeding season when air temperatures are high. Breeding female Southern Yellow-billed Hornbills are confined within a nest cavity to care for the offspring, while males are entirely responsible for provisioning the female and chicks [10]. Male and female hornbills are hence both exposed to challenging thermal environments when breeding.

We investigated whether the beak of this Afrotropical hornbill is functionally similar in terms of heat exchange capacity to that of the Toco Toucan [4]. Following similar methods to those employed by Tattersall et al. [4], we used thermal imaging to quantify heat fluxes in individuals experiencing a range of thermal conditions. We chose birds from a wild study population in the southern Kalahari and examined heat exchange from the beak in comparison to other regions of the body. We predicted that surface temperature of the beak would be regulated so as to promote heat dissipation at air temperatures approaching body temperature, but reduce heat loss under cooler conditions. We further hypothesised that due to differing parental care roles, selection may have favoured sex-specific differences in capacity to use the beak as a thermal radiator.

## Materials and Methods

### Ethics statement

The methods used in this study were approved by the Science Faculty Animal Ethics Committee of the University of Cape Town (protocol number 2013/V24/PR). The study was carried out on private land (Leeupan Guest Farm) with permission of the landowners and of the Northern Cape Department of Environment and Nature Conservation of South Africa (permit number 1166/2013). All bird handling was done by experienced individuals.

### Study species and site

Southern Yellow-billed Hornbills are socially monogamous and widespread in southern Africa, where they occupy savanna and woodland habitats [12]. The individuals used in this study were captured at Leeupan Guest Farm, Northern Cape, South Africa (S 26.95652° E 021.86913°). The study site is in the southern Kalahari Desert where arid savanna dominates the vegetation along a dry riverbed and dune landscape [13]. The site is characterised by cool, dry winters and hot summers with an annual mean rainfall of 215.5 ± 13.0 mm, mean daily summer maximum air temperatures of 34.7 ± 0.05°C. In the last decade, air temperatures exceeded 40°C on 31.1 ± 6.2 days per year during hornbill breeding season (data from the Austral summer months October to March, 1960–2015, Twee Rivieren, 150 km from the study site, South African Weather Service). Temperatures and humidity within tree cavities occupied by a female Southern Yellow-billed Hornbill with one or more chicks at our study site can range between 20.8–43.1°C and 13.8–97.1% relative humidity (van de Ven unpub. data). During incubation and early nestling-rearing female hornbills are confined to the nest cavity, therefore, in contrast to males, they cannot make use of cool microsites within the wider landscape.

### Experimental protocol

In the early austral summer of October 2013, shortly prior to the breeding season, nine adult male and nine adult female hornbills were captured with spring traps (53 x 53 cm) baited with super worms, *Zophobas morio*, and transported in cotton bags to a field laboratory within 4 km of all capture sites. Morphometric measurements were taken from each individual including body mass, wing length, tarsus length, culmen length and maximum culmen height. A lateral-view photograph of the beak and body taken with a DSLR camera (Nikon D3200, Nikon Inc., Melville, U.S.A.) was used to calculate surface areas using ImageJ™, image analysis software (version 1.47, National Institute of Health, United States).

Birds were individually subjected to a ramped profile of increasing air temperatures inside a darkened, custom-built temperature-controlled chamber (1200 x 400 x 300 mm), constructed of corrugated plastic insulated with polystyrene (30 mm thickness). The T_a_ within the cabinet was regulated using a temperature-controlled water bath circulating water through 22-mm diameter copper tubing mounted on the inner wall of the cabinet (design adapted from van de Ven et al. [14]). Air mixing was achieved with a small fan allowing for a uniform T_a_ within the cabinet and fresh air input. Silica gel (500 g) at the bottom of the cabinet prevented increases in humidity via exhaled water vapour and was replaced before becoming saturated. A smaller chamber made of corrugated plastic with an open front and a lid on top (350 x 120 x 350 mm), was placed within the temperature-controlled chamber. Hornbills were placed individually on a perch within the smaller chamber during data collection and would generally remain in this position. Thin nylon netting (17 x 17 mm, 0.2 mm thread thickness) covered the open front of the smaller chamber to prevent the hornbill from moving outside of the field of view of the thermal imaging camera, or coming into contact with the copper piping or silica gel or otherwise injuring themselves. Prior to each experiment, each individual spent 30 minutes habituating to the experimental setup at the initial temperature of 15°C. The T_a_ in the cabinet was increased from 15 to 45°C and held stable at four set point temperatures (15, 25, 35, 45°C). The T_a_ was considered stable when it remained within 2°C of the experimental setpoint for 10 minutes or more. The mean heating rate between each pair of setpoint T_a_ values was 0.7 ± 0.1°C min^-1^. Increasing the temperature in a ramped fashion minimised the time each bird spent in captivity. The T_a_ values we used are within the range that birds naturally encounter in the wild at the study site.

Hornbills spent an average of 120 minutes in the chamber, during which time continuous thermographic images were collected with an infrared camera (ThermoVision A320, FLIR Systems, Danderyd, Sweden) at a frame rate of 15 frames s^-1^. The T_a_ in the chamber was monitored with a NiCr-NiAl thermocouple (Thermocouple HH21A, Omega Engineering, Stamford, U.S.A.) at 5-min intervals, and T_a_ and relative humidity were also recorded with three Thermochron iButtons (DS1923, Maxim, Sunnyvale, CA, USA, resolution = 0.0625°C) at 1-min intervals. The iButtons were calibrated in a circulating water bath against a factory-calibrated NiCr-NiAl thermocouple (Thermocouple HH21A, Omega Engineering, Stamford, U.S.A.). Water vapour pressures (WVP) increased with 0.0289 kPa per 1°C air temperature increment as a consequence of the bird being present in the chamber (S3 Fig). However, the combination of increasing WVP and incremental increases in T_a_ resulted in relative humidity values being approximately constant at 26.6 ± 0.3% during the course of measurements.

The onset of panting in the hornbills was visually assessed from the recorded thermographic image sequence. T_b_ of each hornbill was measured at the start and end of each experiment to assess whether any individuals became hyperthermic during trials. A fine-gauge NiCr-NiAl thermocouple (Thermocouple HH21A, Omega Engineering, Stamford, U.S.A.) was inserted approximately 10 mm into the cloaca, a depth at which a slight withdrawal did not result in a change in the measured T_b_ value. Three males and one female were removed from the chamber early as they became restless at T_a_ < 35°C, giving final sample sizes of 6 males and 8 females at T_a_ > 35°C. All individuals were released at the site of capture immediately after completion of the experiment.

### Data analysis

We assessed how much each region of the hornbill body contributed to overall non-evaporative heat exchange at different T_a_ during the course of the experiment. For each individual, one thermal image per 2.5°C T_a_ increment from 15°C to 45°C was sampled for surface temperature analysis. Preliminary analyses of these images revealed that beak surface temperature typically changed rapidly above a threshold T_a_ value. We averaged the T_a_ where the difference between beak temperature and T_a_ was greatest for the lower mandible, and total beak, for each individual in order to identify the threshold air temperature at which this change occurred (threshold T_a_). Differences in panting and beak threshold T_a_ in response to air temperature between males and females was assessed with a Welch two sample t-test [15].

Surface temperature analysis was done by manually selecting the area of the torso, the gular skin, the lower mandible of the beak and the entire beak in each thermal image using ThermaCAM Researcher Pro 2.9 software (FLIR Systems Inc., Wilsonville). The polygon function in this software allows for accurate selection of the body part of interest and exports the minimum, maximum and mean temperature and the standard deviation of the surface temperature of the selected area. The feet were excluded from this analysis since they were not always visible in the thermal images.

Differences between average body surface temperature (T_s_, component parts: torso, ‘T_s___torso_’`bare gular skin, ‘T_s___skin_’; beak, ‘T_s___beak_’) and air temperature (T_s_-T_a_) were calculated for the different regions of interest across the T_a_ gradient. For all T_s_ except T_s___beak_, we modelled T_s_-T_a_ data using linear mixed models with Gaussian error distribution, T_a_ as the predictor variable and hornbill identity as a random factor. We split the T_s___beak_ -T_a_ dataset below and above the T_s___beak_ threshold T_a_s (lower beak and whole beak) and fitted linear mixed models with Gaussian error distribution to each of the two subsets, again with hornbill identity as a random factor, and T_a_ as the predictor. Linear mixed models were fitted by REML in R 3.1.2 [16] using package lme4 [17]. Normality of all model residuals was confirmed visually using a Normal Q-Q plot [18].

**Fig 1:**
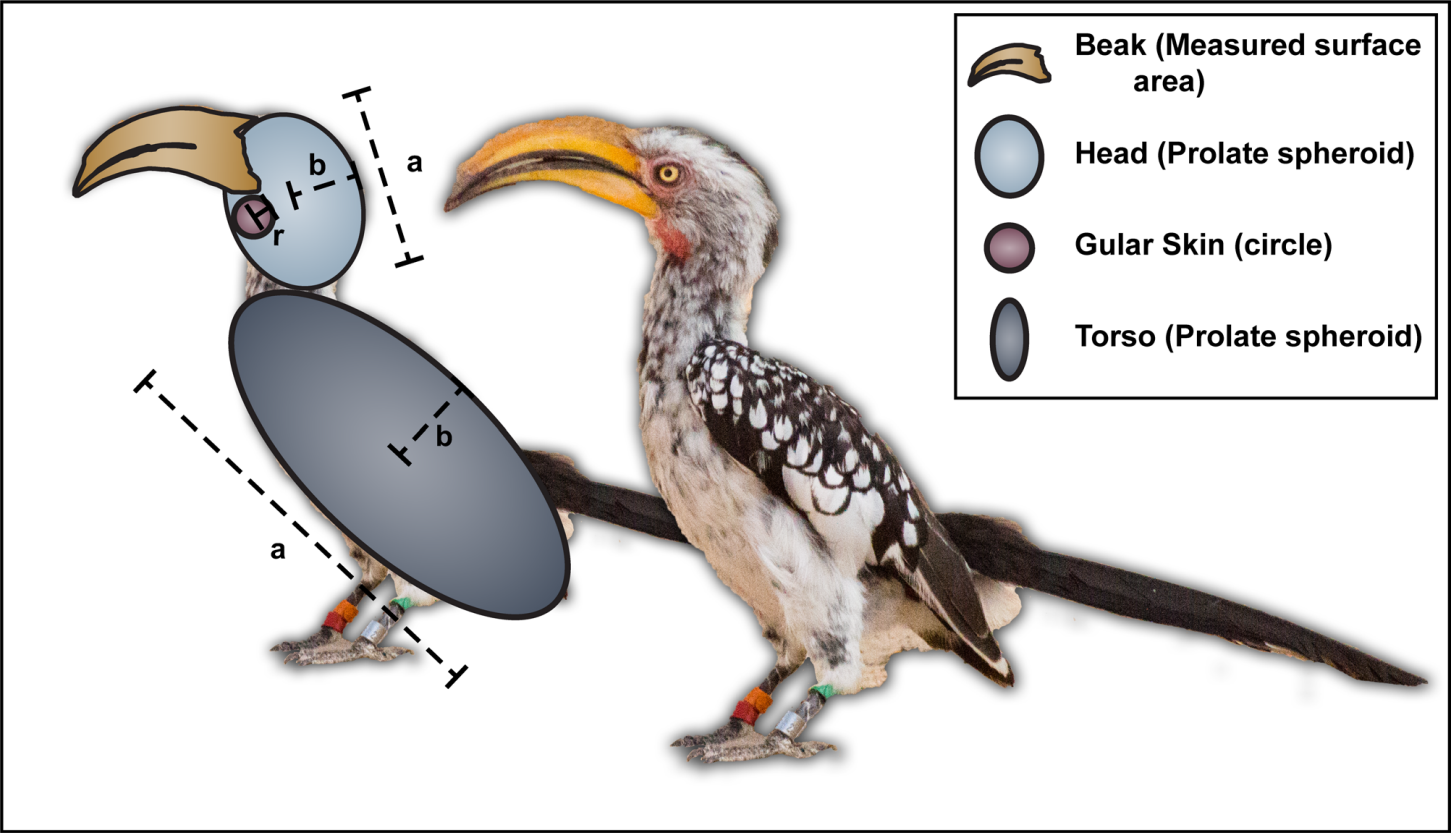
Measurements taken from Southern Yellow-billed Hornbills (*Tockus leucomelas*) to calculate surface areas for estimates of heat transfer.

Morphological measurements from the individuals were modelled according to a geometric model to calculate the body part dimensions and the heat dissipated (Fig 1). The calculated feathered surface area per individual closely matched the predicted relationship of external surface area and body mass as modelled by Walsberg and King [19]. Table 1 gives an overview of the average dimensions of the different parts of the hornbill body.

**Table 1:** Surface area, percentage of total surface area and characteristic dimensions for heat transfer calculated for Southern Yellow-billed Hornbills (*Tockus leucomelas*). n = 18 observations; 9 females and 9 males.

	Surface Area (m^2^)	% Total surface area	Dimension (m)	Nusselt Number
Torso	0.0620 (0.0446–0.0799)	94.9	0.075	Prolate spheroid 3.495
Gular Skin	0.0003 (0.0002–0.0003)	0.4	0.015	Flat circle 3.353
Beak	0.0031 (0.0022–0.0041)	4.7	0.029	Flat polygon 3.566
**Total**	**0.0637**			

The contributions of the different body regions to total heat dissipation were expressed as mean heat dissipation (Watts, W), mean percentage of the total body heat dissipation, and relative heat dissipation (W m^-2^) below and above the beak threshold T_a_. This was done in order to be able to assess the fractional contribution of each body region to total heat exchange, taking into account convective and radiative heat exchange (but not evaporative and conductive heat exchange; calculations following McCafferty et al. [20] and [21], further details S1 Text). These estimates were then used to calculate heat flux per body region as a percentage of the total. Air temperatures above hornbill T_b_ (41.4 ± 0.2°C, data collected from study individuals) were not included in this analysis, because the T_s___beak_ at this stage was cooler than T_a_, resulting in a negative flux value. At these high T_a_ values, the hornbills were observed to pant, indicative of a switch to evaporative water loss as the primary mode of heat dissipation. Linear mixed models with Gaussian error structure were fitted by REML to assess the capacity for heat dissipation in response to T_a_ using package lme4 [17], with individual identity as a random effect. Normality of the model residuals was confirmed visually using a Normal Q-Q plot [16,18].

## Results

**Fig 2:**
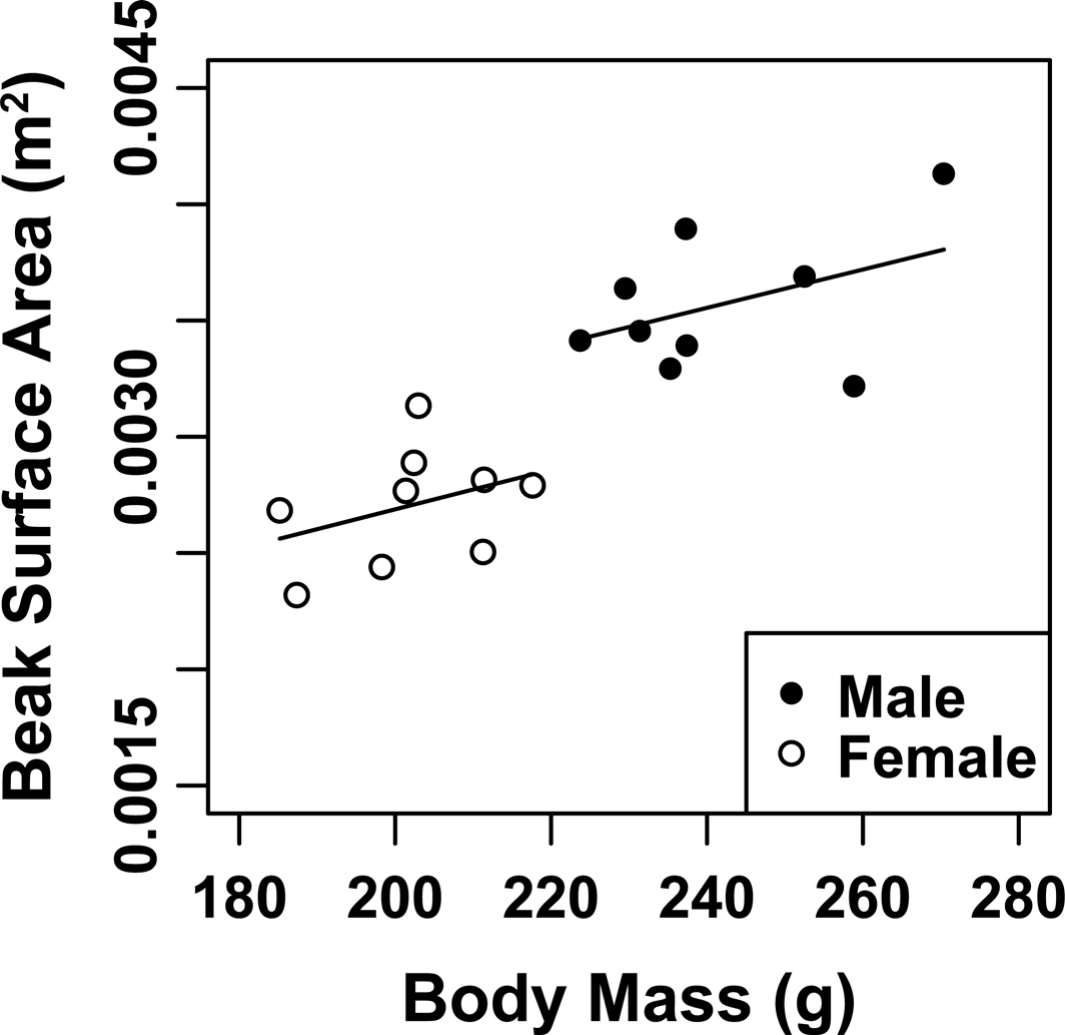
Beak surface area increases as a function of body mass in Southern Yellow-billed Hornbills (*Tockus leucomelas*). Data for nine males and nine females are shown.

**Fig 3:**
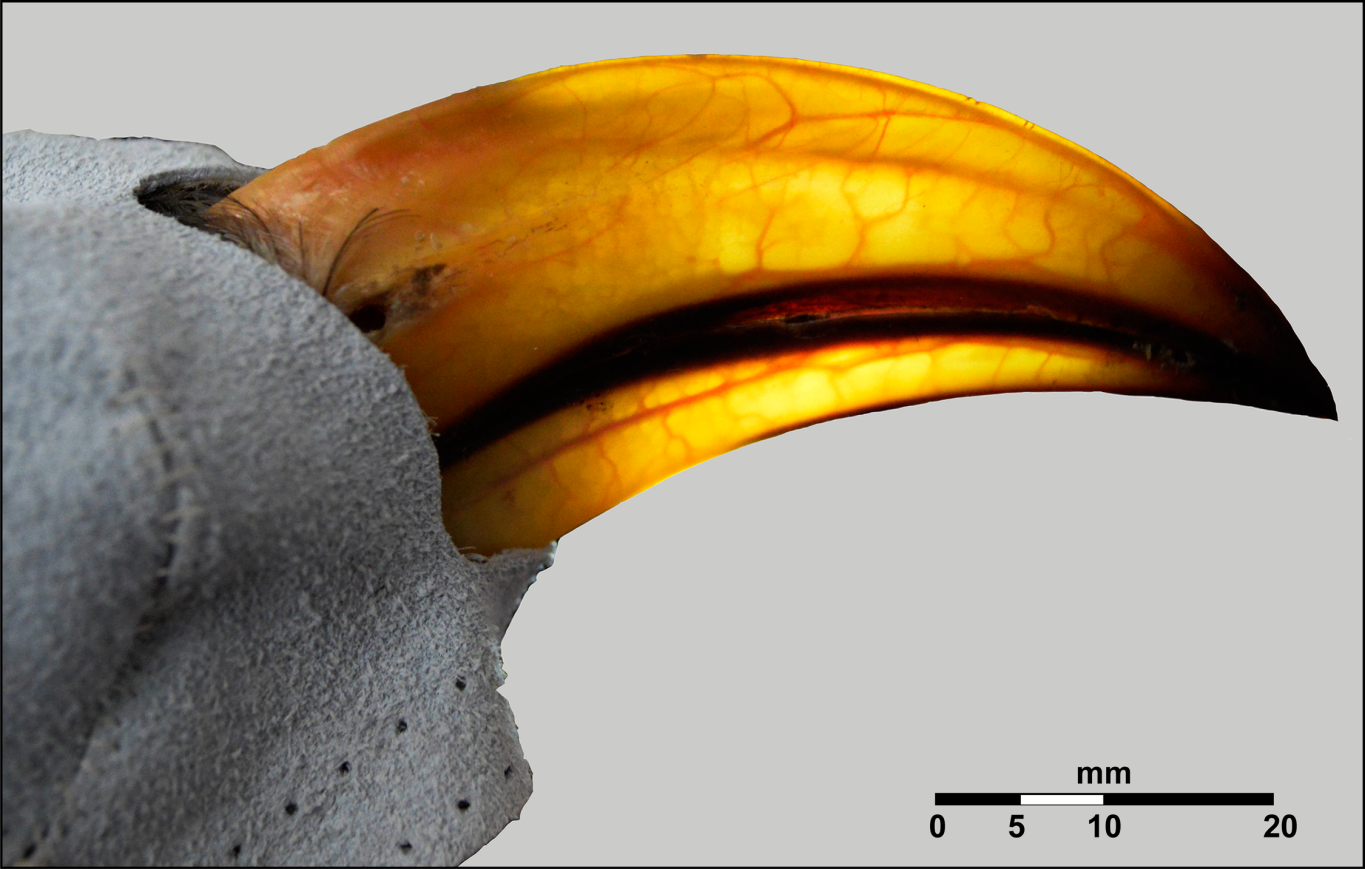
A lateral image of a female Southern Yellow-billed Hornbill (*Tockus leucomelas*) with the beak backlit with a handheld flashlight, revealing the high degree of vascularity.

**Fig 4:**
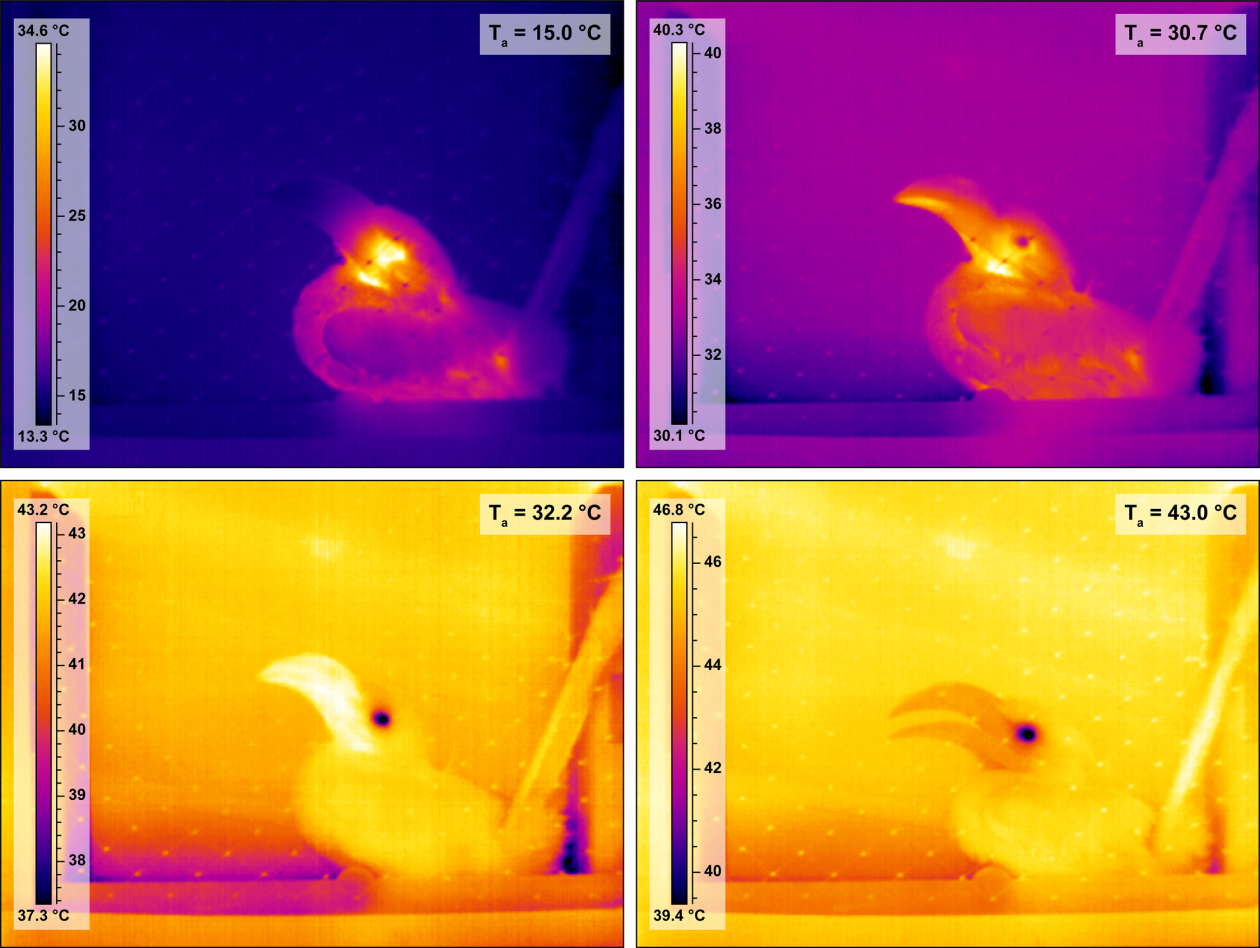
Thermal images of a female Southern Yellow-billed Hornbill (*Tockus leucomelas*) at different air temperatures. Surface temperature (°C) is shown by the scale bar to the left of each image. Top left: the hornbill at air temperature (T_a_) = 15°C: beak surface temperature (T_s___beak_) matches background T_s_. Top right: the hornbill at threshold T_a_ = 30.7°C, T_s_beak_ is changing, lower mandible first. Bottom left: the hornbill at air temperature (T_a_) = 32.2°C, note that T_s___beak_ is much higher than that of the rest of the body and the environment, indicative of heat being radiated from the beak. Bottom right: the hornbill at T_a_ >T_b_ (T_a_ = 43°C). The beak is cooler than the surrounding environment and the bird is using evaporative water loss to keep cool, as indicated by the open beak panting behaviour.

Beak surface area is a sexually dimorphic trait in Southern Yellow-billed Hornbills (Fig 2) and back illumination of the hornbill beak clearly revealed the presence of a network of fine blood vessels below the rhamphotheca in both sexes (Fig 3). As the hornbills were subjected to the ramped profile of increasing air temperature (T_a_), the surface temperature of the beak clearly changed in response to T_a_ (hereafter referred to as threshold T_a_), indicated by a rapid change in the colour of the beak in 14 of the 18 study individuals, as visualised by the thermal imaging camera (mean threshold T_a_ ~30.7°C, Fig 4, S1 Video). Initiation of panting behaviour occurred at T_a_ = 37.4 ± 2.1°C (values are presented as mean ± SE, unless otherwise stated). We found no difference in T_s___beak_ values between males and females at the threshold temperature (Welch two sample t-test: t = 0.61, df = 5.65, p-value = 0.57). Body temperature (T_b_) measurements confirmed that none of the study individuals became severely hyperthermic, with mean T_b_ = 41.4 ± 0.2°C before and 42.2 ± 0.2°C after the experiment.

**Fig 5:**
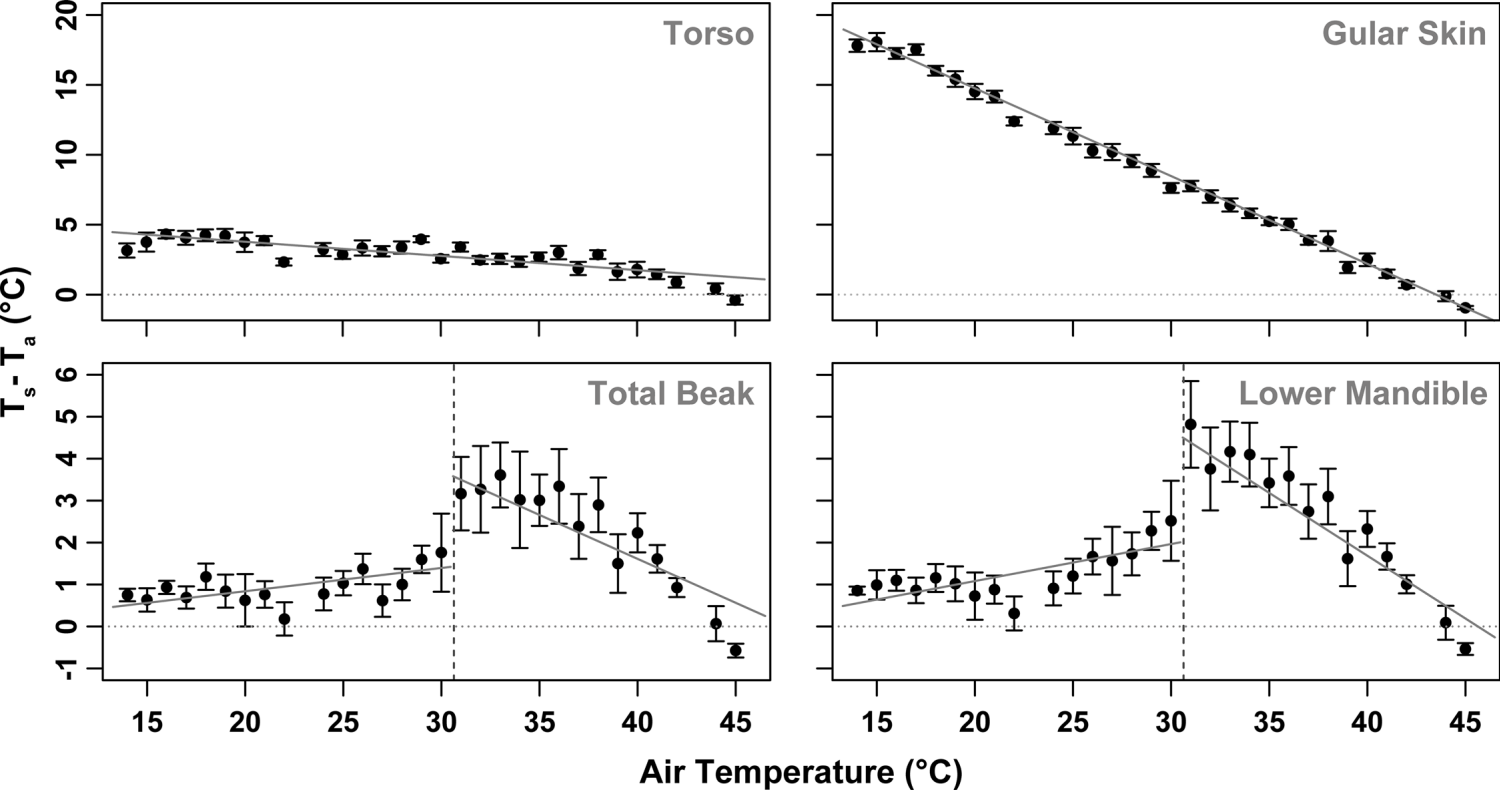
Difference between surface temperature and air temperature (T_s_-T_a_) plotted against air temperature (T_a_) of the torso (T_s___torso_), gular skin (T_s___skin_), the beak as a whole and lower mandible of the beak in Southern Yellow-billed Hornbills (*Tockus leucomelas*). Error bars represent SE. Note that the scaling of the y-axes of the top two panels is different to that of the bottom two panels.

As hypothesised, the relationship between T_s___beak_ and T_a_ differed markedly from that between T_s___skin_ and T_a_ and T_s___torso_ and T_a_. As T_a_ increased from 15°C to 45°C, the difference between skin surface temperature and air temperature (T_s___skin_-T_a_) and the difference between torso surface temperature and air temperature (T_s___torso_-T_a)_ decreased linearly (Fig 5; Table 2). T_s___skin_-T_a_ was just under 30°C when air temperature was close to 15°C. This difference between uninsulated skin temperature and air temperature decreased linearly at a rate of 0.63°C per 1°C increase in T_a,_ as T_a_ approached body temperature. At T_a_ = 45°C, T_s___skin_-T_a_ was below 0°C (ie T_s_skin_ was cooler than T_a_). The rate of change in T_s___torso-_T_a_ with increasing T_a_ was much shallower (-0.11°C per 1°C increase in T_a_); the maximum difference between torso surface temperature and air temperature was 3.9 ± 0.2°C at the lowest experimental temperature (~15°C), likely due to the insulating properties of the feathers (Fig 5; Table 2).

**Table 2:** The relationship between T_s_-T_a_ (°C) and T_a_ (°C), estimates of effect sizes, standard errors (SE), 95% confidence intervals (95% CI) and t-values for Southern Yellow-billed Hornbills (*Tockus leucomelas*). Note the weak response of T_s___beak_-T_a_ to increasing T_a_ at T_a_ < 30.7°C, compared to the strongly negative response T_s___beak_-T_a_ to increasing T_a_ at T_a_ > 30.7°C, Linear mixed models were fitted with Gaussian error distribution and individual bird identity as a random factor. n = 13 observations of 6 males and 8 females.

Variable	Estimate (change in T_s_-T_a_ per 1°C increase in T_a_)	SE	95% CI			t value
Torso (T_s___torso_)	-0.105	0.007	-0.118	-	-0.091	-15.570
Gular skin (T_s___skin_)	-0.634	0.005	-0.645	-	-0.623	-115.690
Beak (T_s___beak_) < T_a_ 30.7°C	0.062	0.018	0.027	-	0.097	3.500
Beak (T_s___beak_) > T_a_ 30.7°C	-0.240	0.035	-0.309	-	-0.172	-6.933

Although variable between individuals, T_s___beak_ -T_a_ was greatest at the threshold T_a_ at which the rapid change in T_s___beak_ occurred. The rapid change of T_s___beak_ was detectable in the lower mandible first at T_a_ = 30.6 ± 1.5°C, followed by the upper mandible at T_a_ = 30.7 ± 1.0°C (Fig 5). Although this sequence (lower mandible followed by upper mandible) was consistent across individuals, inter-individual variation in overall T_s___beak_ -T_a_ thresholds was such that we could find no significant difference between the lower and the upper mandible with respect to the T_a_ threshold at which T_s___beak_ changed (Welch two sample t-test: t = -0.94, df = 31.94, p-value = 0.35). Below the T_s___beak_ threshold temperature, T_s___beak_ -T_a_ increased at a rate of 0.062°C per 1°C increase in T_a_ and at a rate of 0.002 W per 1°C increase in T_a_ (Table 2; Table 3). At T_s___beak_ -T_a_ threshold T_a_ (~30.7°C), T_s___beak_ -T_a_ was maximised and heat was radiated from the beak to the cooler environment with greatest efficiency. As T_a_ increased above this threshold, T_s___beak_ -T_a_ declined at a rate of 0.24°C per 1°C increase in T_a_. At the threshold temperature mean T_s___beak_ -T_a_ was 3.8 ± 0.6°C and the mean heat dissipation from the beak was 0.1 ± 0.0 W: equivalent to 25.1 W m^-2^ (Table 3; Fig 5; S1 Fig). This mechanism of heat dissipation can only be effective over the range of T_a_ from threshold temperature up until T_a_ ≈ T_b_. At T_a_ > T_b_ it is no longer possible for heat to be dissipated passively from the beak to the environment as the temperature gradient is reversed. Reflecting this, when T_a_ > T_b_, T_s___beak_−T_a_ became a negative value reflecting T_b_ (Fig 5). Heat loss per unit surface area via the beak more than doubled at T_a_ > 30.7°C (above the mean T_s___beak_ threshold) compared to T_a_ < 30.7°C (Table 4). Maximum heat dissipation by the beak as a percentage of total body heat dissipation per individual was on average 8.0% (range 1.4–19.9%), and this occurred at mean T_a_ = 32.2°C (range 18.0–39.4°C) (S2 Fig). The maximum percentage of heat loss via the beak was observed at T_a_ = 33.0°C in one individual where the beak at that stage accounted for 19.9% of total heat loss.

**Table 3:** Factors affecting heat dissipation (W), estimates of effect sizes, standard errors (SE), 95% confidence intervals (95% CI) and t-values for Southern Yellow-billed Hornbills (*Tockus leucomelas*). Linear mixed models were fitted with Gaussian error distribution and individual bird identity as a random factor. n = 13 observations of 6 males and 8 females.

Variable	Estimate (change in heat dissipation (W) per 1°C increase in T_a_)	SE	95% CI			t value
Torso (T_s___torso_)	-0.024	0.003	-0.030	-	-0.018	-7.365
Gular skin (T_s___skin_)	-0.002	0.000	-0.002	-	0.002	-63.000
Beak (T_s___beak_) < T_a_ 30.7°C	0.002	0.001	0.001	-	0.003	3.860
Beak (T_s___beak_) > T_a_ 30.7°C	-0.005	0.001	-0.008	-	0.002	-3.485

**Table 4:** Heat dissipation (HD) per body part below and above the threshold temperature for vascular recruitment (30.7°C), as mean heat dissipation (W), percentage of the total body heat dissipation and as relative heat dissipation (W m^-2^) for Southern Yellow-billed Hornbills (*Tockus leucomelas*).

Variable	Mean HD (W)	Mean % HD	W m^-2^
*< 30*.*7°C*			
Torso	1.337	95.189	21.549
Gular skin	0.035	2.509	139.703
Beak	0.032	2.303	10.437
***> 30*.*7°C***			
Torso	0.920	91.082	14.823
Gular skin	0.012	1.228	49.150
Beak	0.078	7.690	25.060

## Discussion

Our data confirm that hornbills, like other birds, can regulate heat exchange from their beaks, using them as thermal radiators when air temperatures are high, but restricting heat loss during cold ambient conditions. The large beaks of both hornbills and toucans are highly vascularised, and control of blood is regulated by vasoconstriction and vasodilation processes [8]. Bird beaks contain branches of major cranial nerves (e.g. the trigeminal nerve) and associated sensory structures [22,23], which require a supply of oxygenated blood. Vascularity of the beak is therefore almost certainly a plesiomorphic avian trait [24]. Heat exchange from the beak occurs in all species investigated to date [1–5,25]. The ability to regulate heat loss via the beak is probably most essential in large-beaked birds because of the need to conserve heat at low temperatures [1,26]. These hornbill beaks therefore function as ‘thermal windows’ [1], similar to better-known examples such as elephant ears [27], bat wings [28], lemur indices [29], dolphin fins [30,31], hummingbird eyes and axial region [32] and bird legs [33–35]. Hughes [11] suggested that the large beaks of toucans and hornbills evolved the ability to facilitate thermoregulation as an exaptation on top of foraging function [11]. However, given the thermal constraints associated with heat loss from a very large beak in the cold, it is also possible that beak size and control over blood flow into the beak evolved in tandem under selective pressure to prevent heat loss during cold periods.

Although both hornbills and toucans have the ability to regulate heat exchange through their large beaks, the threshold temperature for vascular recruitment and relative beak size differed markedly between the two species. Toco Toucans were able to dissipate on average 60% of total heat loss via the beak [4]. The heat dissipated via the beak in Southern Yellow-billed Hornbills was much less: on average ~ 8% of total heat loss (maximum 19.9% at T_a_ of 33.0°C in one individual). We calculated maximum rate per unit surface area under comparable convective conditions using data presented in the figures in the paper by Tattersall et al. [4] and found that toucans dissipated as much as 61.4 W m^-2^ via their beaks, whereas the Yellow-billed Hornbills we studied only dissipated a maximum of 25.1 W m^-2^ via the beak. We suggest three possible explanations for these differences in heat exchange capacity in hornbills and toucans. First, toucans allowed beak surface temperature to rise at considerably cooler air temperature than hornbills (20–25°C compared to 30.5–31°C), allowing them to achieve a steeper gradient between beak surface temperature and air temperature than hornbills, which resulted in a greater capacity for heat dissipation. This difference in threshold temperature may not be an immutable species-specific response. We expect the threshold temperature for vascular recruitment to be phenotypically plastic among populations in response to acclimation to environmental conditions. Second, the rhamphotheca structure of the hornbill beak is twice as hard as that of the toucan beak [36], potentially affecting thermal conductance and the efficiency in heat exchange (D. Andrade pers. comm.). This structural difference in beaks is likely driven by the foraging habits of the two species. In the Kalahari, hornbills use their beaks to break away pieces of tree bark to find invertebrates, a foraging habit that requires a strong beak [10], and is in contrast with beak properties of the toucan, which eats mainly soft fruit [37]. Third, the difference in contribution of the beak to overall heat dissipation between toucans and hornbills could be explained by relative beak size. Positive allometry for beak size occurs in toucans and hornbills, however the Toco Toucan’s beak represents 30–50% of total body surface area, whereas the Yellow-billed Hornbill’s beak only represents 4.7% of its total body surface area. These differences in beak size are perhaps due to demands of diet as explained above: the heavier, thicker rhamphotheca of hornbills and their need for a sturdier beak may preclude the beak achieving the same dimensions as that for toucans. Alternatively, the difference in beak size may reflect the fact that the hornbill is often exposed to air temperatures exceeding body temperature and the uptake of heat via the surface of the beak would be detrimental should the beak be any larger. Allen’s rule [38] predicts a correlation between appendage size and temperature and/or latitude. Large appendages are likely to confer the greatest adaptive benefits, in terms of passive heat loss, to species living in hot environments [2,26,39], with the caveat that they may become a liability when air temperature exceeds body temperature. Hornbills and toucans are distributed across large areas of Africa, Asia and the Americas covering considerable ranges in climate conditions. It would be worth investigating whether within these families, cool climate hornbills and toucans have proportionately smaller beaks than those from hotter climates. One environmental factor that has received relatively little attention as a potential environmental correlate of beak size in birds is humidity. Many species of hornbills and toucans occupy habitats characterised by both high air temperatures and high humidity levels, conditions under which non-evaporative heat dissipation mechanisms are likely to be important. Because of the reduction in evaporative cooling efficiency associated with high water vapour pressures, the capacity to dissipate heat via non-evaporative avenues may, *a priori*, be expected to be under stronger positive selection in humid habitats. This leads to the prediction that thermal radiators such as beaks are more important for species inhabiting humid environments compared to those living in arid areas, an idea first proposed by Greenberg et al. [2]. This might also explain the difference in the threshold temperature at which the birds employ respiratory heat dissipation, toucans pant at 33.1 ± 0.5°C [4] and hornbills at temperatures as high as 37.4 ± 2.1°C. The hornbills have incentive for a high panting threshold temperature in order to conserve water in the arid environment. We suggest that further work on the physiology of heat dissipation through thermal windows, such as large beaks, should include species acclimated to different air temperatures as well as different humidity levels.

In both our study and the study of toucans by Tattersall et al. [4], a few individuals did not display dramatic changes in beak surface temperature as air temperature was increased. In the toucan study, these individuals were juveniles that did not appear to have the capacity to reduce beak surface temperature at low air temperatures [4]. We were unable to determine the age of our study individuals but all appeared to be adults (> 1 year of age [10]). Despite this, four individuals (out of 18) did not exhibit a rapid change in beak surface temperature at any point during the trials: in these individuals beak surface temperature did not differ by more than 2.1°C from air temperature at any point during the entire experiment. We consider that the absence of beak surface temperature change in these individuals could be caused as a result of distress, since peripheral vasoconstriction has been observed in hens (*Gallus gallus domesticus*) in response to a minor discomfort trigger [40].

## Conclusion

Our data add to a growing body of literature revealing the importance of the avian beak in thermoregulation [1–4,6,7,26]. The capacity for non-evaporative heat exchange via the beak appears to be most efficient at air temperatures within ~10°C below body temperature. Beak size in birds is correlated with latitude and air temperature [1]. However, in addition to these we argue that water vapour pressure (and hence the humidity gradient available for evaporative heat loss) in the bird’s habitat, likely gives rise to selection pressure acting on beak size, maximising capacity for radiative and convective heat loss in situations where evaporative cooling is likely to be inefficient. Therefore, we speculate that Allen’s rule [38] may apply to humidity gradients and temperature gradients: large appendages should be particularly advantageous to birds as well as mammals inhabiting hot, but also humid climates. Additionally, beak size may be limited in extreme hot environments due to the risk of heat uptake via the highly vascularised beak when T_a_ exceeds T_b_. We argue that the ability of birds to vasoconstrict the beak at T_a_ > T_b_ would be beneficial to avoid warming up of the blood in the beak, but we were unable to confirm this using thermal imaging data.

## Supporting Information

S1 Data Numerical data. Numerical data used in preparation of Figs 1 and 4; Tables 1, 2, 3 and 4; S1, S2 and S3 Figs.
(XLSX)

S1 Fig Heat loss (Watts) in Southern Yellow-billed Hornbills. Heat loss (Watts) plotted against air temperature (T_a_) of torso, gular skin, the beak and lower mandible of the beak in Southern Yellow-billed Hornbills (*Tockus leucomelas*). Error bars represent SE. Note that the scale of the y-axis of the graphs of gular skin, total beak and lower mandible is different from graph representing the torso, this was done to better illustrate patterns of heat loss by the beak.
(TIF)

S2 Fig Heat loss (% of total) in Southern Yellow-billed Hornbills. Heat loss as a proportion of total body heat loss (%) plotted against air temperature (T_a_) of torso, gular skin, the beak as a whole and lower mandible of the beak in Southern Yellow-billed Hornbills (*Tockus leucomelas*). Data above the panting initiation temperature (T_a_ = 37.4 ± 2.1°C) has not been included in this graph since evaporative heat loss has not been assessed and this makes total heat loss after initiation of panting incomplete.
(TIF)

S3 Fig Relative humidity (%) and water vapour pressure (kPa) in the temperature cabinet. Relative humidity (%) and water vapour pressure (kPa) in the temperature cabinet in response to air temperature (°C). Data are combined from all the individual experiments. Error bars represent SE.
(TIF)

S1 Text Additional methods for heat transfer calculation (DOCX)

S1 Video Thermal imaging sequence of the Southern Yellow-billed Hornbill. Thermal imaging sequence of the Southern Yellow-billed Hornbill (*Tockus leucomelas*) during the experiment.
(MP4)
