# Supplementary figures and images for: Regulation of Heat Exchange across the Hornbill Beak: Functional Similarities with Toucans?
Source: PLoS One. 2016 May 18;11(5):e0154768. doi: 10.1371/journal.pone.0154768 (PMC4871549; doi:10.1371/journal.pone.0154768)

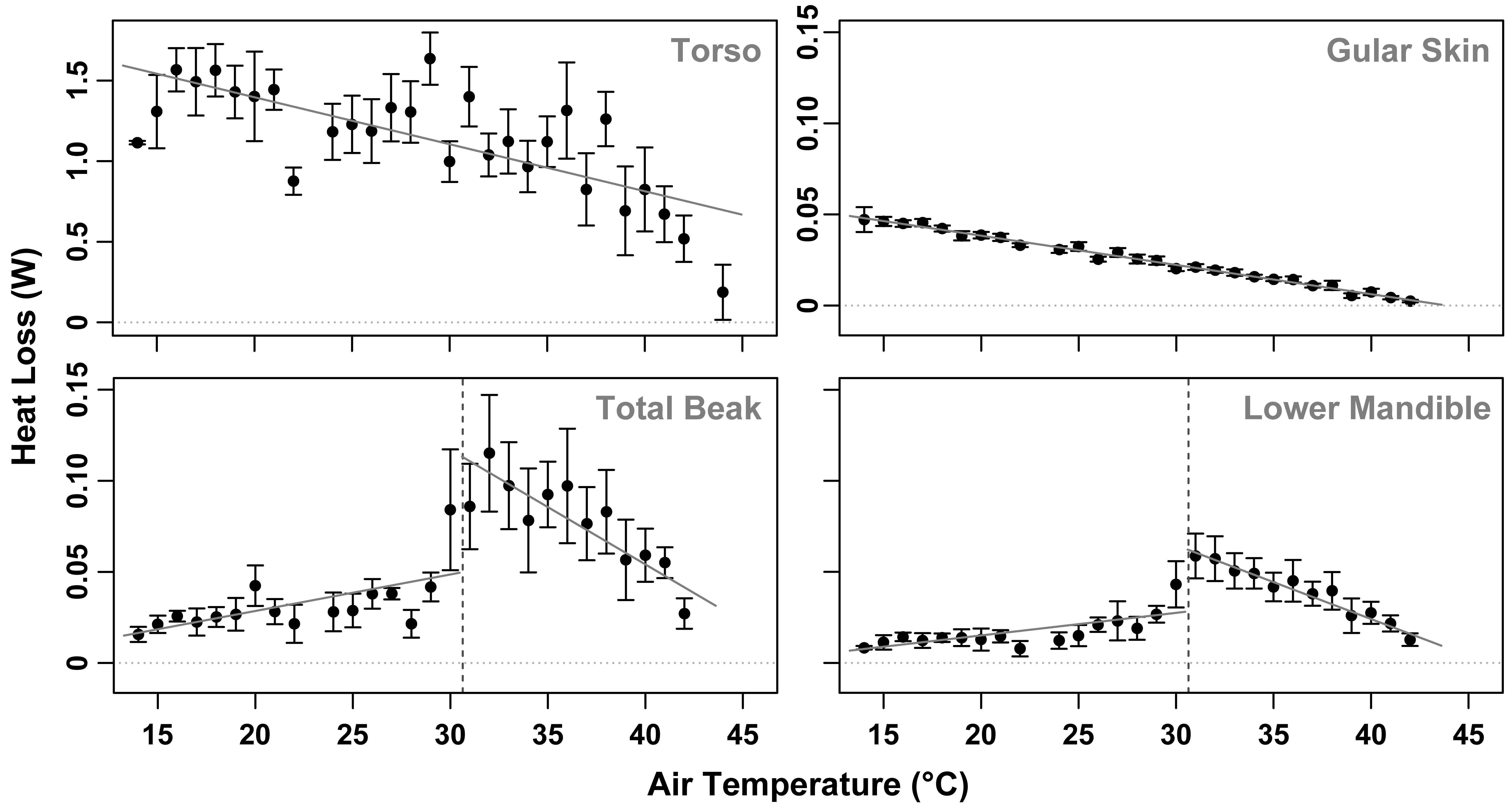

Supplement: S1 Fig — Heat loss (Watts) plotted against air temperature (Ta) of torso, gular skin, the beak and lower mandible of the beak in Southern Yellow-billed Hornbills (Tockus leucomelas). Error bars represent SE. Note that the scale of the y-axis of the graphs of gular skin, total beak and lower mandible is different from graph representing the torso, this was done to better illustrate patterns of heat loss by the beak. (TIF) [file pone.0154768.s002.tif]

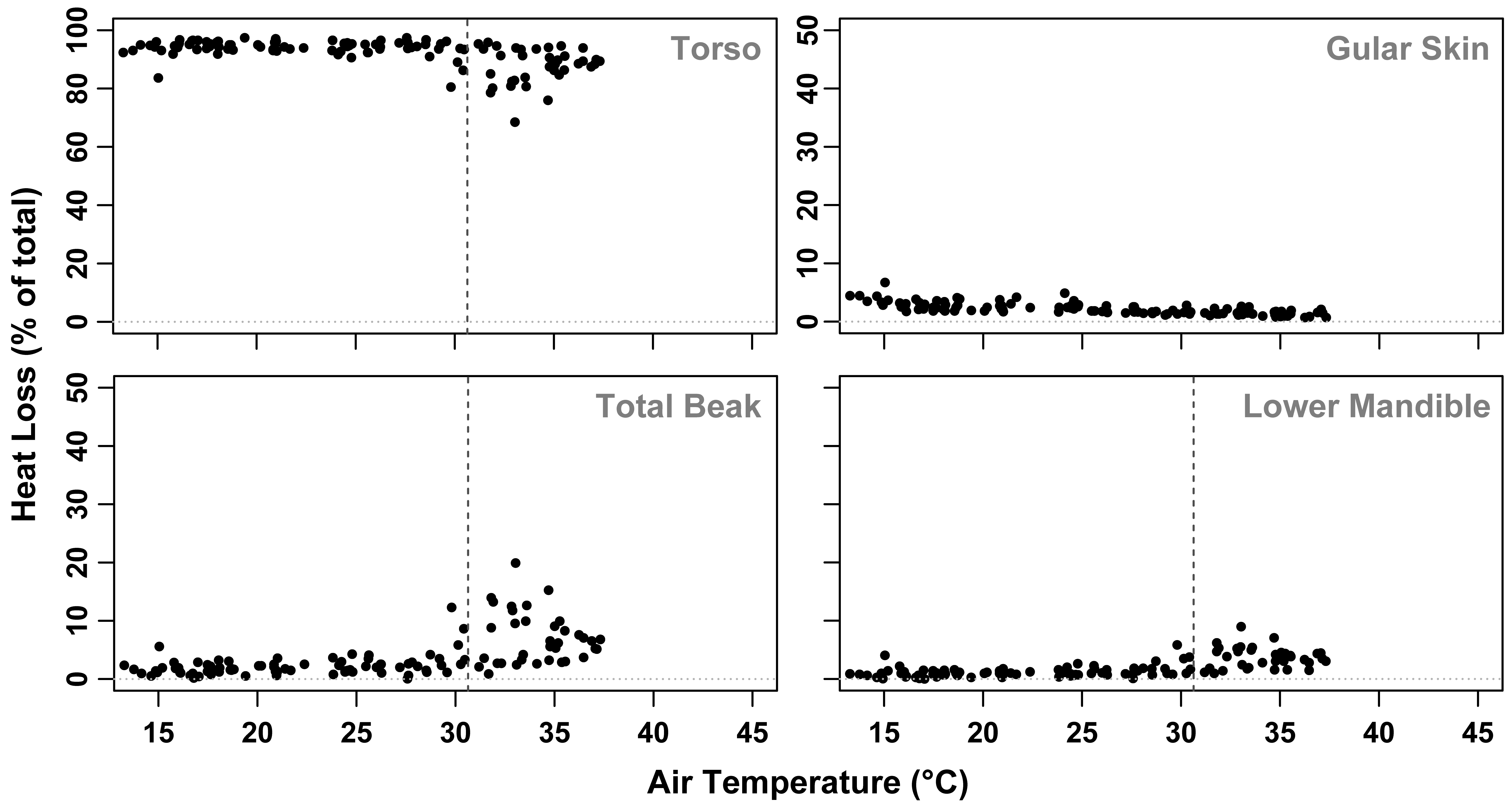

Supplement: S2 Fig — Heat loss as a proportion of total body heat loss (%) plotted against air temperature (Ta) of torso, gular skin, the beak as a whole and lower mandible of the beak in Southern Yellow-billed Hornbills (Tockus leucomelas). Data above the panting initiation temperature (Ta = 37.4 ± 2.1°C) has not been included in this graph since evaporative heat loss has not been assessed and this makes total heat loss after initiation of panting incomplete. (TIF) [file pone.0154768.s003.tif]

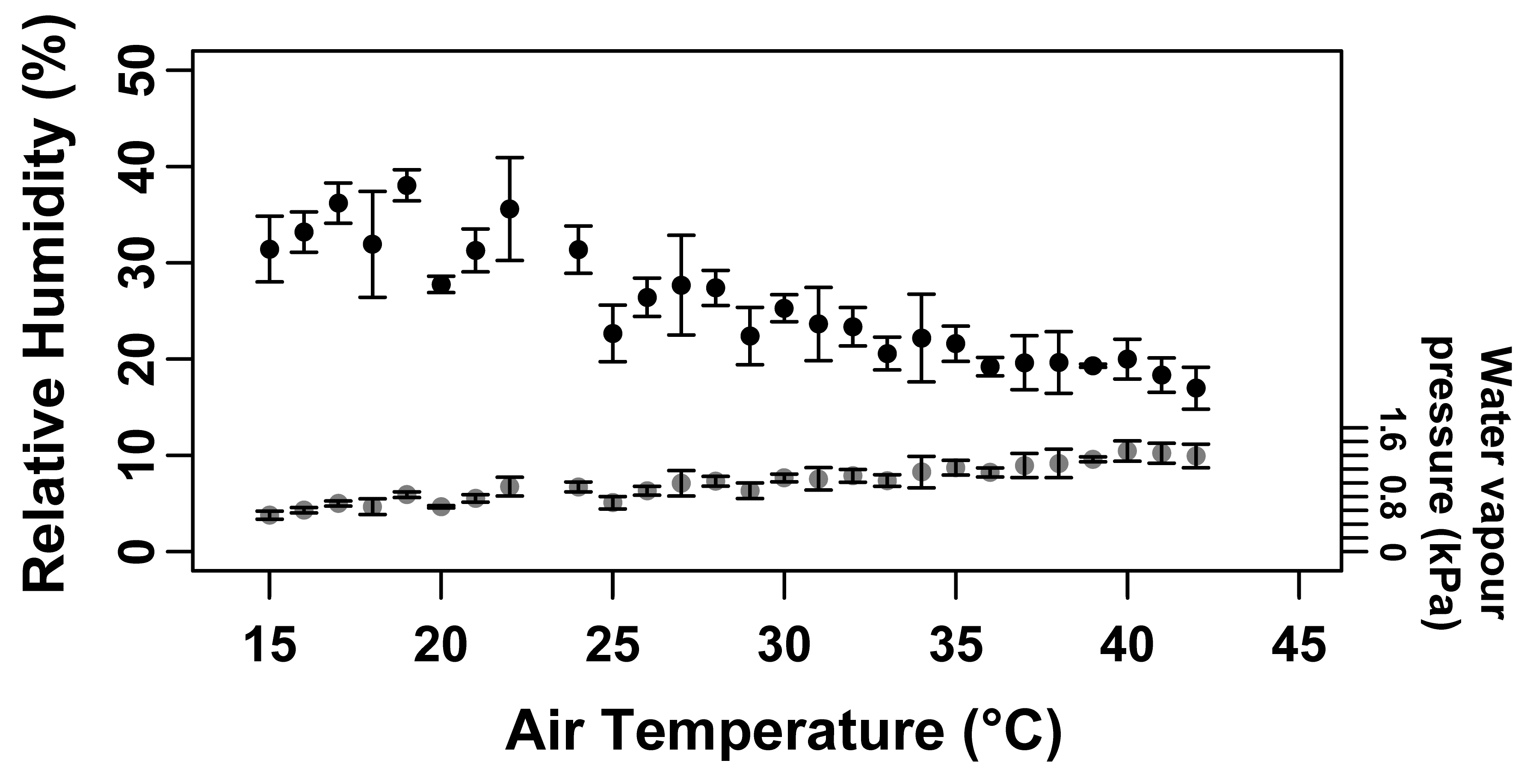

Supplement: S3 Fig — Relative humidity (%) and water vapour pressure (kPa) in the temperature cabinet in response to air temperature (°C). Data are combined from all the individual experiments. Error bars represent SE. (TIF) [file pone.0154768.s004.tif]
