# Supplementary material for: Regulation of Heat Exchange across the Hornbill Beak: Functional Similarities with Toucans?
Source: PLoS One. 2016 May 18;11(5):e0154768. doi: 10.1371/journal.pone.0154768 (PMC4871549; doi:10.1371/journal.pone.0154768)
Supplement: S1 Text — (DOCX) [file pone.0154768.s005.docx]

## S1 Text. Additional methods for heat transfer calculation

In controlled conditions radiative heat transfer takes place from the bird to the environment and from the wall of the chamber to the environment. Radiative heat exchange can be calculated for each of the body parts of the bird with the following formula:

Where *A* (m^-2^) represents the surface area of each body part, σ is the Stefan-Boltzmann constant (5.67 x10^-8^ Wm^-2^K^-1^), ɛ_a_ is the emissivity of bird plumage (0.95) and ɛ_w_ is the emissivity of the white plastic walls of the bird chamber (0.84). T_s_ and T_w_ are the radiative surface temperature of the surface of the body part and the surface of the wall (°K). A small fan in the chamber provided the bird with fresh air and prevented a decrease in oxygen levels and an anemometer recorded air flow in the chamber during the experiment. The movement of air caused by the fan did not create a measurable air flow and therefore convective heat transfer occurred via free convection and was calculated as:

Whereby *A* (m^2^) represents the surface area of each body part, T_s_ is the surface temperature of the body part (°K) and T_a_ is the air temperature (°K). h_c_ is the convective heat transfer coefficient and can be calculated as follows:

k represents the thermal conductivity of air, determined at each T_a_ (W m^-2^ °K^-1^), d (m) is the characteristic dimension of each body part and Nu is the dimensionless Nusselt number. The Nusselt number is a measure of the ratio of buoyant to viscous forces and is dependent on the shape of the characteristic body part [1]. In order to measure the conductive heat loss, the temperature difference between the bird and any object it touches needs to be taken into account. During this experiment the bird body only came into contact with the perch via the feet. We ignored conductive heat loss, because of the low conductivity of wood (0.12 W m^-2^K^-1^) and small surface contact of the feet on the perch. We did not attempt to measure evaporative heat loss in this study, but instead we recorded the chamber temperature at which the bird initiated panting behaviour. Panting, or buccopharyngeal evaporative water loss, coincides with other evaporative routes like cutaneous and cloacal heat loss [2]. The total heat loss from the hornbill body was therefore only calculated below the panting initiation temperature as the sum of the radiative and conductive heat transfer:

## References

1. Monteith J, Unsworth MH (2013) Principles of Environmental Physics: Plants, Animals, and the Atmosphere. Amsterdam: Academic Press.

2. Hoffman TC, Walsberg GE, DeNardo DF (2007) Cloacal evaporation: an important and previously undescribed mechanism for avian thermoregulation. J Exp Biol 210: 741-749.
